# TNFRSF1B Signaling Blockade Protects Airway Epithelial Cells from Oxidative Stress

**DOI:** 10.3390/antiox13030368

**Published:** 2024-03-18

**Authors:** Javier Checa, Pau Fiol, Marta Guevara, Josep M. Aran

**Affiliations:** Immune-Inflammatory Processes and Gene Therapeutics Group, Genes, Disease and Therapy Program, Institut d’Investigació Biomèdica de Bellvitge—IDIBELL, 08908 L’Hospitalet de Llobregat, Spain; jcheca@idibell.cat (J.C.); pfiol@idibell.cat (P.F.); mguevaraf@idibell.cat (M.G.)

**Keywords:** oxidative stress, airway epithelial cells, cystic fibrosis, RNAi knock-down, TNFRSF1B

## Abstract

Progressive respiratory airway destruction due to unresolved inflammation induced by periodic infectious exacerbation episodes is a hallmark of cystic fibrosis (CF) lung pathology. To clear bacteria, neutrophils release high amounts of reactive oxygen species (ROS), which inflict collateral damage to the neighboring epithelial cells causing oxidative stress. A former genome-wide small interfering RNA (siRNA) screening in CF submucosal gland cells, instrumental for mucociliary clearance, proposed tumor necrosis factor receptor superfamily member 1B (*TNFRSF1B*; *TNFR2*) as a potential hit involved in oxidative stress susceptibility. Here, we demonstrate the relevance of TNFRSF1B transcript knock-down for epithelial cell protection under strong oxidative stress conditions. Moreover, a blockade of TNFR signaling through its ligand lymphotoxin-α (LTA), overexpressed in airway epithelial cells under oxidative stress conditions, using the anti-tumor necrosis factor (TNF) biologic etanercept significantly increased the viability of these cells from a toxic oxidizing agent. Furthermore, bioinformatic analyses considering our previous RNA interference (RNAi) screening output highlight the relevance of *TNFRSF1B* and of other genes within the TNF pathway leading to epithelial cell death. Thus, the inhibition of the LTα3-TNFR2 axis could represent a useful therapeutic strategy to protect the respiratory airway epithelial lining from the oxidative stress challenge because of recurrent infection/inflammation cycles faced by CF patients.

## 1. Introduction

Cystic fibrosis (CF) is a monogenic disease caused by the dysfunction of the cystic fibrosis transmembrane conductance regulator (CFTR), an ATP-gated anion channel located at the apical side of epithelial cells, leading to multiorgan affectation [1,2]. Nevertheless, the lung becomes mostly affected and, indeed, respiratory failure is the main cause of mortality among CF patients. In fact, the inability to carry out a proper electrodiffusion of not only Cl^−^ but also HCO_3_^−^ and other small anions impairs electrolyte balance in the epithelial lining of the airways. This leads to mucus dehydration and thickening which, in turn, prevents ciliary beating, severely affecting lung physiology [3,4]. This environment is prone to bacterial infection and colonization, leading to progressive biofilm formation and concomitant chronic inflammation. However, it is still unclear whether the immune-inflammatory process occurring in the airways is a direct consequence of CFTR dysfunction or results from confronting bacterial clearance. Certainly, the innate immune system plays a key role in the fight against pathogen infection. Particularly, phagocytic cells such as macrophages and neutrophils, attempting to clear infection, release high amounts of reactive oxygen species (ROS) to an already high oxidative environment, which inflict collateral damage to the airway epithelial cells [5,6]. Furthermore, the consequences of such chronic insult are aggravated by the fact that cells holding dysfunctional CFTR are unable to release the key natural defense antioxidant glutathione [7]. Therefore, the establishment of efficient mechanisms conferring the increased protection of the airway epithelia from the toxic oxidative milieu owing to repeated infection/inflammation flares should improve the outcome of CF lung disease.

In a previous study, we performed a genome-wide RNA interference (RNAi) screening using a randomized small interfering RNA (siRNA) library to identify oxidative stress susceptibility genes/pathways in CF submucosal gland epithelial cells [8]. We unveiled the involvement of the tumor necrosis factor (TNF) pathway in oxidative stress and turned to a particular screening hit, the tumor necrosis factor receptor superfamily member 1B (*TNFRSF1B*; *TNFR2*) gene (Gene ID: 7133) [9]. Here, we show that the TNF signaling pathway, which holds a key role in several physiological and pathological processes including cell proliferation, cell death, and inflammation [10,11], is also involved in oxidative stress in airway epithelial cells. We demonstrate that siRNA-mediated TNFRSF1B transcript knock-down or TNFR signaling blockade through etanercept, a soluble TNFR2-IgG1 Fc fusion protein, can confer oxidative stress resistance in CF airway epithelial cells. This might become therapeutically relevant to extend lung performance or to prevent lung function decline in CF patients.

## 2. Materials and Methods

### 2.1. Cell Culture

Both 16HBE14o- human bronchial epithelial cells and 6CFSMEo- CF human submucosal gland epithelial cells were kindly provided by Dieter Gruenert (UCSF, San Francisco, CA, USA) [12] and maintained in Minimum Essential Medium (MEM) with Earle’s salts (Gibco; Thermo Fisher Scientific, Waltham, MA, USA). These cell lines were supplemented with 2 mM glutamine, penicillin (100 U/mL), streptomycin (100 µg/mL), and 10% fetal bovine serum (FBS) (all from Gibco) and were grown at 37 °C and 5% CO_2_.

### 2.2. Cloning of Anti-TNFRSF1B siRNA

**Figure 1:**
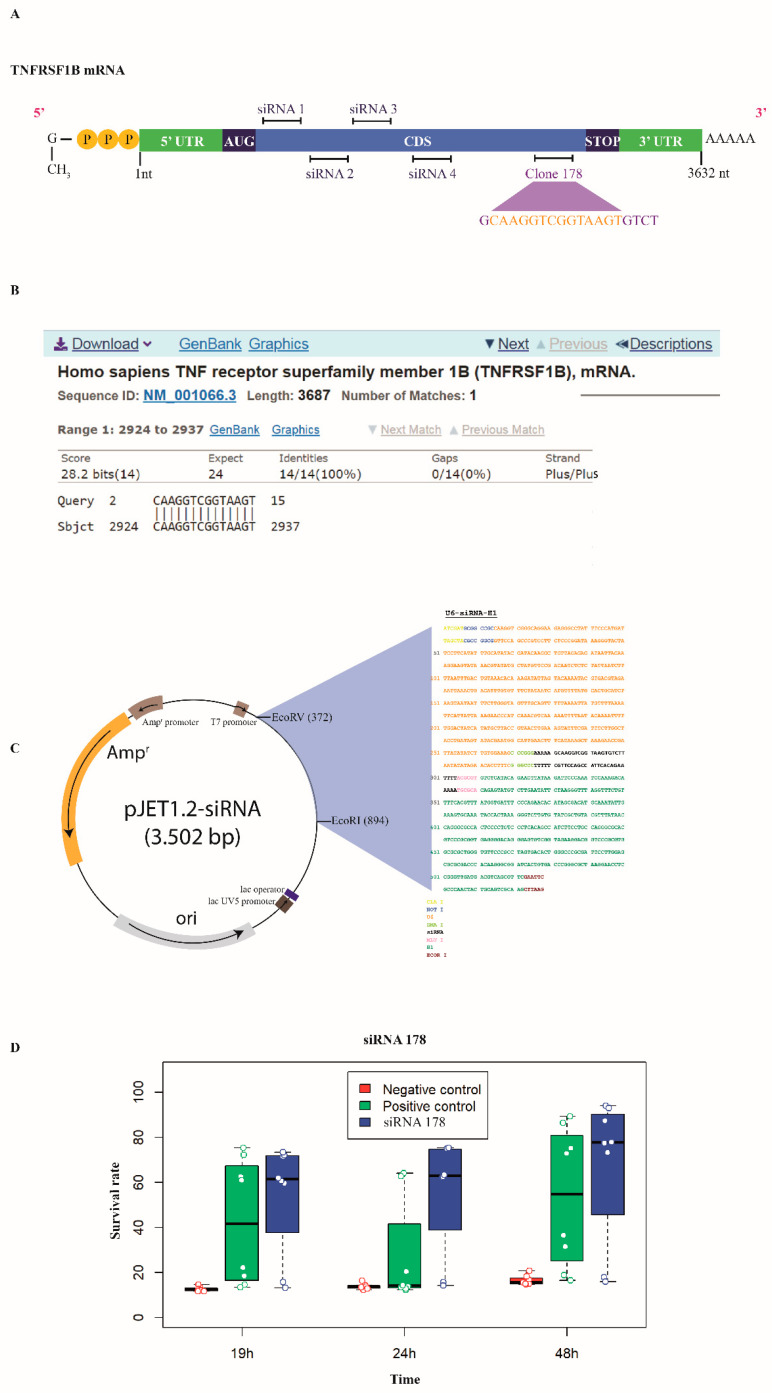
*TNFRSF1B* is involved in oxidative stress in airway epithelial cells. (**A**) A schematic representation of the TNFRSF1B transcript showing the relative positions targeted by the four optimized siRNA sequences comprising the ON-TARGET plus SMART pool against TNFRSF1B and the sequence from clone 178, obtained by whole-genome RNAi screening. (**B**) Clone 178 shows 73% homology to TNFSF1B, according to BLAST analysis (sequence highlighted in yellow in (**A**)). (**C**) A convergent U6/H1 RNA pol III promoter cassette containing clone 178 was transferred to the pJET1.2 cloning vector generating pJET1.2-siRNA. (**D**) pJET1.2-siRNA was introduced into 6CFSMEo- airway epithelial cells by reverse transfection, and these cells were exposed to a lethal dose of 0.3 mM H_2_O_2_. The graphic shows the survival rate of 6CFSMEo- cells (clone 178, in blue) compared with that of a negative control siRNA (in red) and a positive control siRNA (clone 171, in green), at 19 h, 24 h, and 48 h after the addition of the Alamar Blue viability reagent. n = 8 replicates/sample.

The siRNA sequence against TNFRSF1B (clone 178; Figure 1A) was previously obtained through a high-throughput RNAi screening using a randomized siRNA library aimed to identify oxidative stress susceptibility genes [8]. The referred sequence introduced within the human U6 and H1 RNA polymerase III promoters arranged in opposite orientations [13] was amplified by proofreading the polymerase chain reaction (PCR) through “ClaI/NotI F” and “EcoRI-H1 R” primers (Table 1), cloned into the pJET1.2 vector (Thermo Fisher Scientific) obtaining pJET1.2-siRNA (Figure 1), and sequence verified (STAB VIDA, Caparica, Portugal) using “pJET 1.2 F” and “pJET 1.2 R” primers (Table 1).

### 2.3. Reverse Cell Transfection and Short-Term Selection

A reverse transfection protocol, where freshly passaged cells are added to pre-plated transfection complexes, was employed to assess the capacity of the anti-TNFRSF1B siRNA to confer oxidative stress resistance. Briefly, 200 ng of pJET 1.2-siRNA plasmid DNA were mixed with 2 μL TransIT-2020/μg DNA per well of a 96-well plate. The corresponding transfection complexes formed after 45 min met with 2 × 10^5^ cells per well added dropwise, in a final volume of 220 μL. This mixture was removed after 48 h, and the wells were refilled with an equal volume of 0.3 mM H_2_O_2_ [8]. After 6 h of cell exposure to H_2_O_2_, the oxidant was removed, 100 μL/well of Alamar Blue (AB) reagent (Bio-Rad, Hercules, CA, USA) was included, and three spectrophotometer readings were performed at 19 h, 24 h, and 48 h to monitor cell viability (PowerWave XS, BioTek, Winooski, VT, USA). Cells reverse transfected with pEGFP-N1 (Clontech, Mountain View, CA, USA) were employed as a negative control.

### 2.4. Differential Gene Expression Analysis

The airway epithelial cells under study underwent total RNA extraction (RNeasy Mini Kit; Qiagen, Hilden, Germany), and the relevant transcripts were subsequently measured by reverse transcription (RT)-qPCR employing the High-Capacity cDNA Reverse Transcription Kit, the corresponding inventoried TaqMan Gene Expression Assays, and the thermal cycler QuantStudio 5 Real-Time PCR System (all from Thermo Fisher Scientific). For mRNA abundance quantification, the relative fold change was inferred employing the ΔΔCt method (2^−ΔΔCt^ equation) and peptidylprolyl isomerase A (PPIA; Gene ID: 5478; Accession #: NM_021130) as the endogenous reference transcript.

### 2.5. Validation of siRNA-Mediated Knock-Down of TNFRSF1B Expression

siRNA pools (ON-TARGET plus siRNAs in SMART pool format, Dharmacon, Lafayette, CO, USA) directed against the TNFRSF1B transcript were verified in both 6CFSMEo- and 16HBE14o- cells by transient transfection following the manufacturer’s recommendations. Thus, ON-TARGET plus siRNAs in SMART pool format or ON-TARGET plus Non-targeting siRNA (C-) (3.4 μL of 2.0 μM; Dharmacon, Lafayette, CO, USA; Table 1): TransIT-X2 (7.5 μL; Mirus Bio, Madison, WI, USA) complexes were produced for 20 min at room temperature in 250 μL of non-supplemented MEM and added dropwise to semiconfluent (60–80%) wells from a 6-well plate during 48 h until subsequent analyses.

### 2.6. Bioluminescent Reporter Assay

A quantitative luciferase reporter assay using the pmirGLO Dual-Luciferase miRNA target expression vector according to the manufacturer’s indications (Promega, Madison, WI, USA) validated the silencing efficacy of Dharmacon’s siRNAs against TNFRSF1B.

Primary human monocytes were chosen for RNA extraction because these immune cells express the TNFRSF1B transcript at significant levels. Total blood from a healthy donor was acquired from the Blood and Tissue Bank (Barcelona, Spain) under informed consent and promptly processed (within 16 h after extraction) to fractionate peripheral blood mononuclear cells (PBMCs) by Ficoll-Paque density centrifugation (GE Healthcare Bio-Sciences AB). Monocytes were isolated by affinity through magnetic mouse anti-human CD14 monoclonal antibody-conjugated microbeads (MACS, Miltenyi Biotec, Auburn, CA, USA), and its purity (>90%) was assessed by CD14 staining and flow cytometry (FACSCanto II flow cytometer including FACSDiva software v 6.1.3 (Becton Dickinson, Franklin Lakes, NJ, USA)). After RNA isolation and RT, a 592 bp TNFRSF1B region was chosen for PCR amplification, containing three of the four siRNA Dharmacon target sequences. The employed forward and reverse primers against the TNFRSF1B cDNA (TNFR2 F and TNFR2 R) included the NheI and the SalI restriction sites, respectively (Table 1). The resulting PCR fragment was then inserted into the corresponding NheI/SalI restriction sites of the pmirGLO vector, generating the pmirGLO-TNFRSF1B vector.

Transfections were performed in wells from a 96-well plate, where pre-made complexes (5 μL/each of lipofectamine (Thermo Fisher Scientific) plus pmirGLO-TNFRSF1B DNA (100 ng) for 15 min at room temperature) and cells (3 × 10^5^ cells in 75 μL) were added sequentially. The following day, an analogous transfection was repeated replacing the pmirGLO-TNFRSF1B plasmid with siRNA (50 nM). Negative control samples contained no siRNA. At 24 h after siRNA transfection, the Firefly luciferase activity was recorded using the substrate Dual-Glo Luciferase reagent. In the following 15 min, the Renilla luciferase activity was measured using the Dual-Glo Stop & Glo substrate, both in a luminometer (Fluostar optima; BMG Labtech, Ortenberg, Germany). Relative activity was expressed as the Firefly/Renilla ratio.

### 2.7. Cell Death Induction Trough Oxidative Stress

H_2_O_2_ was employed as the source of oxygen-derived free radicals to induce oxidative stress in airway epithelial cells. The performance of anti-TNFRSF1B siRNAs or the TNF antagonist etanercept to confer oxidative stress resistance was assessed in the airway epithelial cell lines.

To evaluate the effect of the siRNAs, 5 × 10^3^ of cells per well was added in a 96-well plate to a final volume of 100 μL/well. After 24 h, the anti-TNFRSF1B siRNAs were incubated at the previously indicated concentrations for 24 h, according to preliminary optimization assays. Consequently, the cells were exposed to 1.2 mM H_2_O_2_ plus fresh siRNAs. As a negative control, cells treated with a non-targeting siRNA (siRNA(C-)) and exposed to 1.2 mM H_2_O_2_ were used.

To assess the outcome of etanercept (Enbrel^®^, Pfizer, New York, NY, USA), 2 × 10^5^ 6CFSMEo- or 16HBE14o- cells per well was added to a 96-well plate in a final volume of 220 μL/well. At 46 h, etanercept was incubated for 2 h at a concentration of 25 μg/mL for 16HBE14o- and 50 μg/mL for 6CFSMEo- cells. Next, 220 μL/well of 0.3 mM H_2_O_2_ was added. As a negative control, cells without etanercept treatment were analyzed.

In both cases, after 6 h of cell exposure to H_2_O_2_, the oxidant was removed and 100 μL/well of AB reagent was added for cell viability monitorization. Spectrophotometric readings were made at the indicated times and according to the manufacturer’s instructions.

### 2.8. Identification of Putative siRNA Targets

The blastn algorithm (NCBI BLAST) [14] identified candidate siRNA targets by sequence homology, searching against human genomic and transcript databases, employing highly similar sequence optimization (megablast) [15], adjusting for short input sequences, and retrieving a maximum of 250 hits per query sequence. The output generated involved >63% of siRNA sequences covered by the target. List hits included mRNA, RNA, or protein molecules with accession number prefix NM, NR, NP, XM, XR, or XP. The annotations file comprising gene symbols and Entrez Gene identifiers was extracted from https://ftp.ncbi.nih.gov/gene/DATA/ (accessed on 27 May 2020).

### 2.9. Pathways Overrepresentation Analysis (ORA)

Through the clusterProfiler R package (v3.14.3) [16], an enrichment analysis (ORA) of the gene lists of interest was performed and independently computed over Kyoto Encyclopedia of Genes and Genomes (KEGG) knowledgebase [17] considering the Entrez Gene identifiers. Using a hypergeometric distribution test, a *p*-value was obtained for each interrogated biological pathway or GO term. The background distribution was defined by all available annotations in the knowledgebase. PathwAX (https://pathwax.sbc.su.se/ (accessed on 16 June 2021) [18] was employed for network crosstalk-based pathway annotation. The Benjamini–Hochberg method was employed to control the False Discovery Rate (FDR) in multiple hypothesis testing [19].

### 2.10. Statistical Analysis

The capacity to confer resistance to oxidative stress was assessed for the siRNA sequence targeting the TNFRSF1B transcript (siRNA 178) obtained in the initial screen through descriptive statistics and using a mixed-effects model, as previously described [8].

For all other data, normality distribution was evaluated through the Shapiro–Wilk test and, consequently, group differences were assessed using parametric (*t* test, analysis of variance (ANOVA)) or non-parametric (Wilcoxon, Friedman) tests, corrected for multiple comparisons by the Holm–Sidak method. Data are given, unless otherwise stated, as mean values (mean ± SD). A value of *p* < 0.05 is considered statistically significant.

## 3. Results

### 3.1. The Involvement of the TNF Pathway and the TNFRSF1B Gene in Oxidative Stress in CF Airway Epithelial Cells

A high-throughput RNAi screening for oxidative stress susceptibility genes acting in CF airway epithelial cells yielded 167 siRNA sequences able to induce H_2_O_2_-mediated oxidative stress resistance in 6CFSMEo cells and 4452 putative targets with unique Entrez identifiers (Table S1, see “Material and Methods” for details) [8]. Thus, we focused on a particular siRNA sequence targeting the TNFRSF1B (TNFR2) transcript (Figure 1A) according to the following evidence: (i) this siRNA presented a high percentage of continuous matching homology with the *TNFRSF1B* gene (73%, according to Basic Local Alignment Search Tool (BLAST) analysis) (Figure 1B); (ii) its function as a putative death receptor [10,20,21] and the implication of the TNF-TNFR pathway as an important hub regulated by ROS in inflammation [22].

We aimed to evaluate the silencing activity of the anti-TNFRSF1B siRNA, included in the convergent U6/H1 RNA pol III promoter-containing plasmid pJET1.2-siRNA (Figure 1C), to confer oxidative stress resistance in airway epithelial cells. A fast functional assay through reverse transfection confirmed the ability of this siRNA to prevent 6CFSMEo- cell death under a lethal concentration of H_2_O_2_ (Figure 1D).

**Figure 2:**
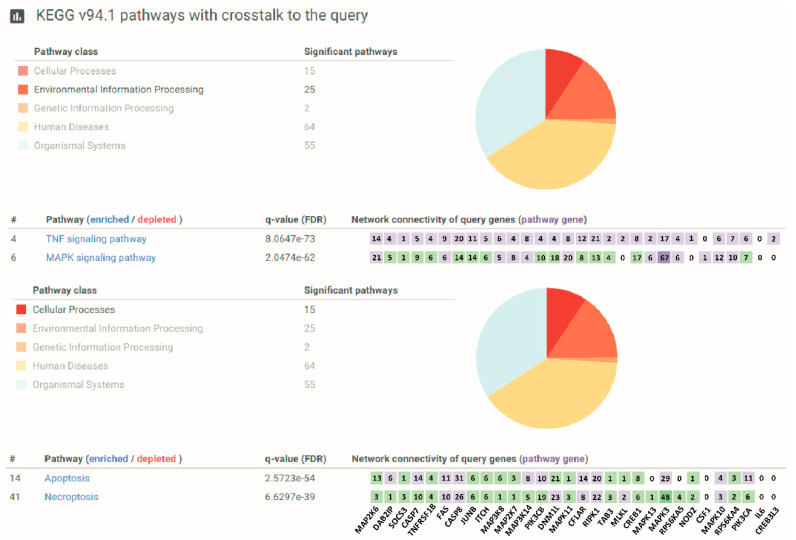
Both TNF-related and cell death pathways are engaged in oxidative stress in airway epithelial cells. Pathway annotation analysis based on the crosstalk between the 31 gene set obtained by enrichment analysis over the KEGG knowledge base (see Table 1) and FunCoup (PathwAX II; https://pathwax.sbc.su.se/ (accessed on 16 June 2021). The most significant pathways sorted by the increased FDR within the “Environmental Information Processing” class (**top image**) and the “Cellular Processes” class (**bottom image**) are shown. The genes linked to a given pathway are depicted as green squares, while the genes that are part of that pathway are depicted in violet. The degree of network connectivity for each of the query genes is also shown as a number within each square and ranges from 0 to 67.

Indeed, a gene enrichment analysis computed over the KEGG knowledgebase identified a set of 31 oxidative stress susceptibility genes (potential siRNA targets) involved in the TNF signaling pathway (hsa04668) (Table 2). Furthermore, a pathway annotation analysis based on crosstalk between our 31 gene set and FunCoup, a framework for genome-wide functional association networks [18], unveiled the relevance of these siRNA target genes within the TNF and mitogen-activated protein kinase (MAPK) (hsa04010) signaling pathways (top two most significant pathways within the “Environmental Information Processing” class) and in apoptosis and necroptosis (top two most significant pathways within the “Cellular Processes” class) (Figure 2).

### 3.2. TNFRSF1B Transcript Knock-Down Induces Resistance to Oxidative Stress in Airway Epithelial Cells

To substantiate the above findings, the performance of the anti-TNFRSF1B siRNA sequence transcribed from pJET1.2-siRNA, obtained by high-throughput screening, was compared with that from an siRNA pool of four different anti-TNFRSF1B siRNA sequences targeting different non-overlapping positions located within the coding sequence of the TNFRSF1B transcript (Figure 1A).

The TNFRSF1B knock-down efficacy of the commercial siRNA pool was confirmed through an indirect luciferase assay. A 592 bp fragment of the TNFRSF1B transcript including three of the four siRNA pool target sequences was placed downstream of the Firefly luciferase gene within the pmirGLO vector (Figure S1A,B) and overexpressed in 6CFSMEo- cells. Thus, the siRNA pool was able to achieve up to 80% reduction in TNFRSF1B expression with this system (Figure S1C).

**Figure 3:**
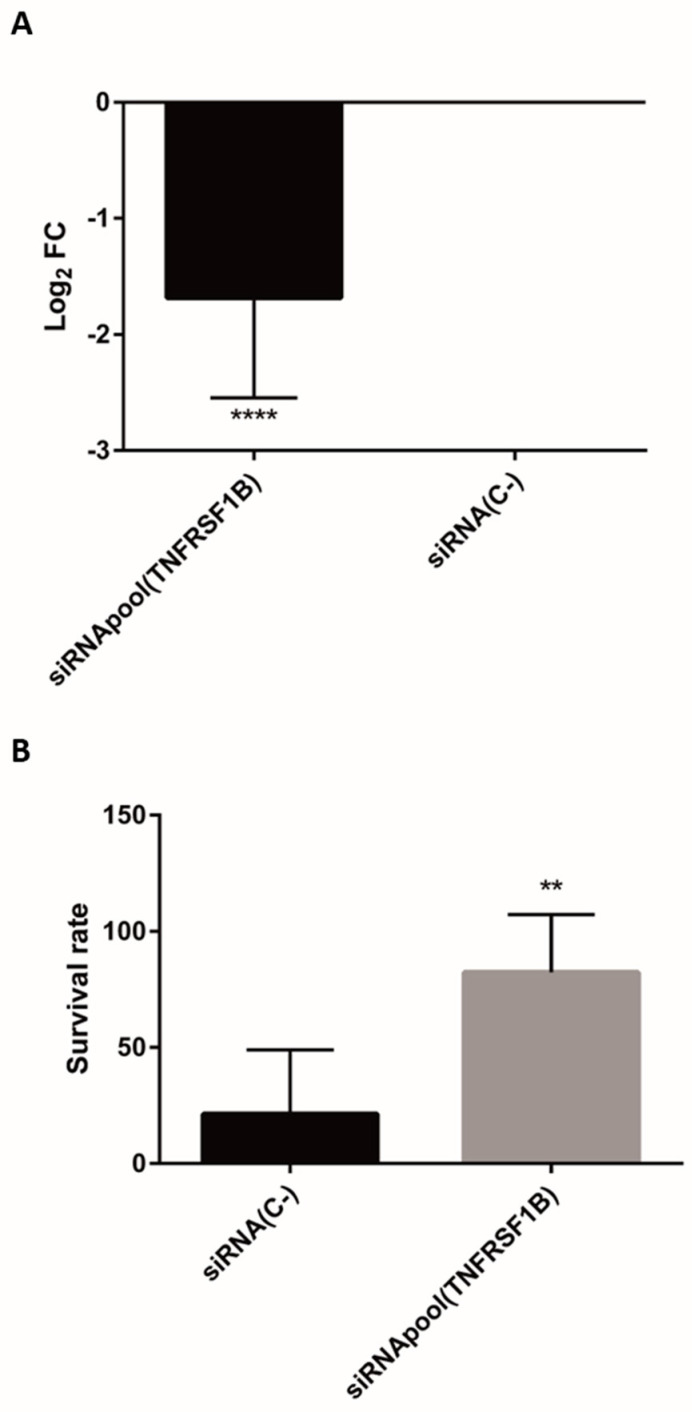
An optimized siRNA pool targeting the TNFRSF1B transcript reduces its mRNA levels and increases cell survival in airway epithelial cells under oxidative stress conditions. (**A**) Log_2_FC values of 16HBE14o- cells transfected with the ON-TARGET plus SMART pool siRNA against TNFRSF1B, compared to those transfected with a control ON-TARGET plus Non-targeting siRNA (siRNA(C-)). Data are expressed as means ± SD. n = 10, with three technical replicates/each. (**B**) The quantification by spectrophotometry of the mean survival rate of the control ON-TARGET plus Non-targeting siRNA (siRNA(C-)) and the ON-TARGET plus SMART pool siRNA against TNFRSF1B in 16HBE14o- cells at 4 days after the addition of 1.2 mM H_2_O_2_ and the Alamar Blue viability reagent. Data are expressed as means ± SD. n = 3 with eight technical replicates/each. ** *p* < 0.01, **** *p* < 0.0001, compared to the control siRNA(C-).

Additionally, parallel transfections of 6CFSMEo- [8] and 16HBE14o- (this study) airway epithelial cells were performed to assess the effect of the optimized siRNA pool targeting the endogenous TNFRSF1B transcript. A substantial silencing effect induced by the siRNAs was evident in both cell lines, revealed by significantly reduced relative TNFRSF1B mRNA levels when analyzed by RT-PCR ([8] and Figure 3A). To correlate the significant downregulation of TNFRSF1B mediated by siRNA silencing with oxidative stress resistance, the viability of both 6CFSMEo- and 16HBE14o- airway epithelial cells under a near-lethal H_2_O_2_ concentration was assessed. Increased cell survival induced by RNAi against TNFRSF1B was evidenced under strong oxidative stress conditions in both CF and non-CF epithelial cells, confirming the involvement of this receptor in oxidative stress and respiratory pathophysiology ([8] and Figure 3B).

### 3.3. The Levels of Lymphotoxins (LTs) Are Modulated by Oxidative Stress in Airway Epithelial Cells

**Figure 4:**
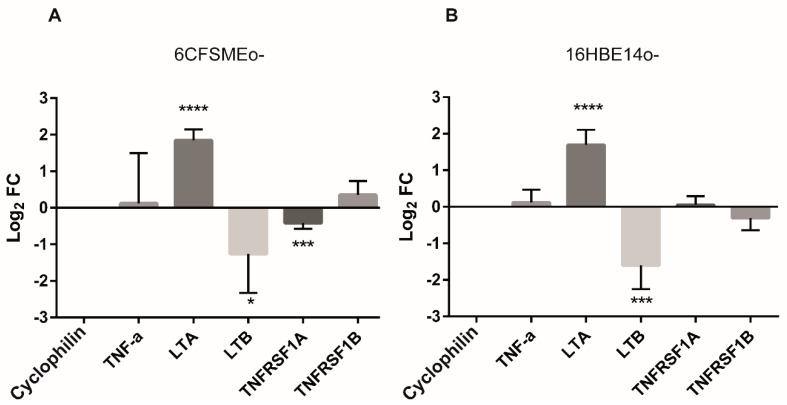
LTA is upregulated under oxidative stress conditions in airway epithelial cells. Log_2_FC values of TNF receptors (TNFRSF1A and TNFRSF1B) and related ligands (TNF-α, LTA, and LTB) in 6CFSMEo- (**A**) and 16HBE14o- (**B**) cells after 1 h exposure to 0.3 mM H_2_O_2_. Transcript levels were measured by RT-qPCR. Data are expressed as means ± SD. n = 4, with two technical replicates/each. * *p* < 0.05, *** *p* < 0.001, and **** *p* < 0.0001 compared to the control cyclophilin A.

Preliminary RT-qPCR analyses indicated that *TNFRSF1B* was a low-abundance gene constitutively expressed in airway epithelial cells in normal conditions and that transfection procedures did not perturb its expression in these cells. Thus, we further inquired whether the relative expression levels of the main ligands and receptors of the TNF/LT axis could be affected under oxidative stress conditions in both 6CFSMEo- and 16HBE14o- cells. Interestingly, TNF-α and its corresponding TNFRs (TNFRSF1A and TNFRSF1B) levels were undisturbed, while LTA was significantly upregulated and, conversely, LTB was significantly downregulated after oxidative stress induction (Figure 4). Consequently, increased soluble LT-α3 homotrimer levels because of oxidative stress interacting with TNFRs could drive the death of airway epithelial cells.

### 3.4. The Anti-TNF Biologic Etanercept Increases the Cell Survival of Airway Epithelial Cells under Oxidative Stress Conditions

**Figure 5:**
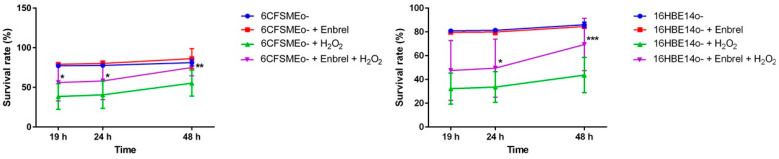
Etanercept induces oxidative stress resistance in airway epithelial cells. The spectrophotometric quantification of the mean survival rate of 6CFSMEo- and 16HBE14o- airway epithelial cells under strong oxidative stress conditions at 19 h, 24 h, and 48 h after adding etanercept and the Alamar Blue viability reagent. Etanercept was added at 25 μg/mL for 16HBE14o- cells and at 50 μg/mL for 6CFSMEo- cells. Furthermore, the cells were stressed with 0.3 mM H_2_O_2_. Data are expressed as means ± SD. n = 5 (6CFSMEo-); n = 4 (16HBE14o-), with four technical replicates/each. * *p* < 0.05, ** *p* < 0.01, and *** *p* < 0.001 compared to the control cells without etanercept treatment.

The biologic etanercept (Enbrel^®^), a fusion protein between TNFR2 and IgG1 Fc, acts as a decoy receptor for TNFs. We assessed whether the blockade of LT-α as TNFR1 and TNFR2 ligand could lead to the same outcome as the siRNA-mediated knock-down of TNFRSF1B expression under oxidative stress in airway epithelial cells. Therefore, exposing different airway epithelial cell lines (6CFSMEo- and 16HBE14o-), preincubated with etanercept, to strong oxidant conditions (Figure 5) prevented its apoptotic/necrotic fate.

Taken together, the above results consistently suggested that the biologic etanercept might be protective against oxidative stress processes, inducing a significant improvement in cell survival in the airway epithelium.

## 4. Discussion

Through functional genomics, we have shown here a relevant role of TNFRSF1B in oxidative stress-induced cell death occurring in airway epithelial cells and, particularly, in CF submucosal gland cells. Certainly, a high-throughput RNAi screening has revealed the direct involvement of TNFRSF1B (TNFR2) but not of TNFRSF1A (TNFR1), the other known TNF receptor, and multiple downstream transcripts along the TNF pathway (Figure S2) in oxidative stress.

Furthermore, we have validated the importance of TNFR2 in airway epithelial cell physiology through specific RNAi-mediated TNFR2 transcript knock-down, which conferred oxidative stress resistance in these cells. It has been shown that TNFR2 can be indirectly involved in apoptotic pathways and inflammatory signal transduction [23,24,25].

We have shown the absence of TNFα transcript upregulation in airway epithelial cells under oxidative stress. Conversely, the significant induction of the alternative TNFR ligand transcripts LTA (towards upregulation) and LTB (towards downregulation) was evident in these H_2_O_2_-stressed cells. LTα can exist as soluble homotrimeric LTα3 or as two transmembrane heterotrimeric complexes termed LTα1β2 and LTα2β1 [26,27]. Yet the LTα3 trimer seems to be the biologically active autocrine secreted form causing apoptosis/necrosis in oxidant-stressed airway epithelial cells through interaction with TNFR1 and/or TNFR2 [28,29]. It has been shown that LTα3 induces apoptosis, necroptosis, and inflammatory signals with the same potency as TNFα [30]. Moreover, cell death could be augmented by the specific activation of TNFR2, being a result of crosstalk, where the activity of TNFR2 indirectly influences the TNFR1 signaling complexes [24]. Therefore, when levels of ROS are high, cell death might be triggered through the LT-α3-TNFR2 axis, which would recruit the cytosolic complex TNFR-associated factor 2 (TRAF2)-cellular inhibitor of apoptosis protein 1/2 (cIAP1/2), preventing its interaction with TNFR1-associated death domain protein (TRADD). Free TRADD would then join Fas-associated death domain (FADD) and procaspase 8, leading to receptor-interacting serine/threonine protein kinase (RIPK)-mediated apoptosis/necroptosis.

Besides RNAi targeted against the TNFR2 transcript, an alternative approach to interfere with the LTα3-TNFR2 axis and, consequently, preserve airway epithelial cell integrity under strong oxidative stress conditions would involve the blockade of TNFR2 function through anti-TNF biologics. Among them, etanercept is a biologic immunomodulator generated through the fusion of the extracellular moiety of the TNFR2 receptor and the Fc domain of human IgG1 and blocks the activities of both TNFR1 and TNFR2 ligands. Thus, etanercept reduces the inflammation associated with some diseases including rheumatoid arthritis, psoriatic arthritis, severe axial spondyloarthritis, and mild or severe psoriasis as an alternative to other ineffective treatments [31]. Accordingly, our results indicate that etanercept interferes with the TNF pathway and, particularly, with TNFR2 signaling preventing apoptosis and, conversely, increasing cell survival, which would have a positive impact on airway epithelial cell regeneration after an oxidative stress episode. To note, etanercept has not shown dose-dependent or target organ toxicity. Thus, it has a good safety profile for clinical use and might become useful for the treatment of oxidative stress-related pathologies such as CF. Remarkably, the therapeutic efficacy of etanercept (Enbrel^®^) in CF patients has been demonstrated in two separate clinical case studies. As reported, etanercept resolved their rheumatoid arthritis but, most importantly, improved their lung function and reduced their sputum neutrophils, leading to fewer pulmonary infective exacerbations requiring hospitalization [32,33].

Of note, this proof-of-concept study has two potential limitations. Firstly, the heterogeneity and plasticity of the respiratory epithelium is well known, with common and rare epithelial cell types in different sections of the respiratory tract [34], thus our results employing two particular cell types may not be fully extrapolatable due to the inherent complexity of the airways. Secondly, the relevance of the TNFR2 blockade for oxidative stress protection should be further validated in well-differentiated primary CF and non-CF airway epithelial cell cultures, which more closely recapitulate the in vivo morphology and pathophysiology of human airway epithelia.

## 5. Conclusions

The TNF pathway seems to play an important role in mediating oxidative stress processes in airway epithelial cells. Our results indicate that etanercept could be effective in preventing the LTα3-TNFR2 interaction, reducing apoptosis, and increasing airway epithelial cell survival, particularly within the chronic hyperoxidant environment of the CF airways. Thus, our study along with the above-mentioned clinical proof-of-concept reports support the feasibility of etanercept repurposing for the prevention/treatment of CF pulmonary exacerbations pending further validation through clinical trials.

## Figures and Tables

**Table 1:** Primer sequences for anti-TNFRSF1B siRNA cloning and validation, and pre-designed siRNAs directed against the TNFRSF1B transcript (Accession #: NM_001066).

Primer Sequences			
Name	Sequence (5′ → 3′)		Number of nt
ClaI/NotI F	CCGCGCATCGATGCGGCCGCCAAGGTCGGGCAGGAAGAGGGCCTA		45
EcoRI-H1 R	CGCGAATTCGAACGCTGACGTCATCAACCCGCTCCAAGGAATCGC		45
pJET 1.2 F	CGACTCACTATAGGGAGAGCGGC		23
pJET 1.2 R	AAGAACATCGATTTTCCATGGCAG		24
TNFR2 F	GCGCGCTAGCTAGTTCGGGAACAGAACCGCATC		33
TNFR2 R	GCGCGTCGACTAGTGGCCTTATCGGCAGGCAAGT		34
**Pre-Designed siRNAs (TNFRSF1B)**			
**siRNA Name**		**Sequence (5′ → 3′)**	
ON-TARGET plus Non-targeting siRNA (C-)		UGGUUUACAUGUCGACUAA	
ON-TARGET plus SMART pool siRNA J-003934-05 (siRNA1)		CGACUUCGCUCUUCCAGUU	
ON-TARGET plus SMART pool siRNA J-003934-06 (siRNA2)		GGAAUGUGCCUUUCGGUCA	
ON-TARGET plus SMART pool siRNA J-003934-07 (siRNA3)		CAUCAGACGUGGUGUGCAA	
ON-TARGET plus SMART pool siRNA J-003934-08 (siRNA4)		AGCCUUGGGUCUACUAAUA	

**Table 2:** Oxidative stress susceptibility transcripts involved in the TNF signaling pathway obtained through RNAi screening.

Target Symbol (Accession #)	Target Entrez	siRNA id *	Homology (%) *
DAB2IP (NM_138709)	153090	39/66/132	63/63/73
RPS6KA4 (NM_003942)	8986	146/172	63/73
TNFRSF1B (NM_001066)	7133	178	73
SOCS3 (NM_003955)	9021	139	63
NOD2 (NM_022162)	64127	161	68
RPS6KA5 (NM_182398)	9252	6	68
MAP2K7 (NM_145185)	5609	29	68
CASP8 (NM_001228)	841	122	78
IL6 (NM_000600)	3569	32	89 (+1 mismatch)
CSF1 (NM_000757)	1435	132	73
MAP3K14 (NM_003954)	9020	8/136	73/63
PIK3CA (NM_006218)	5290	176	68
MAPK11 (NM_002751)	5600	141	73
MAPK3 (NM_002746)	5595	54	68
MAPK10 (NM_002753)	5602	20/26/147	73/63/63
MAP2K6 (NM_002758)	5608	75/112	63/63
CFLAR (NM_003879)	8837	113	63
FAS (NM_000043)	355	119	63
CASP7 (NM_001227)	840	124	63
RIPK1 (NM_003804)	8737	125	63
MLKL (NM_152649)	197259	146	63
JUNB (NM_002229)	3726	148	63
CREB3L3 (NM_032607)	84699	149	63
ITCH (NM_031483)	83737	153	63
PIK3CB (NM_006219)	5291	27/56	63/68
MAP3K8 (NM_005204)	1326	52	63
DNM1L (NM_012062)	10059	73	63
LIF (NM_002309)	3976	8	73
MAPK13 (NM_002754)	5603	83	63
TAB3 (NM_152787)	257397	89	63
CREB1 (NM_004379)	1385	96	63

* The same transcript sequence can be the target of different siRNAs with different percentages of homology.

## Data Availability

Data are contained within the article and Supplementary Material.

## Supplementary Materials

The following supporting information can be downloaded at: https://www.mdpi.com/article/10.3390/antiox13030368/s1, Figure S1: Optimized siRNAs induce the efficient RNAi-mediated knock-down of TNFRSF1B. (A) A PCR-mediated amplification of a 605 bp DNA portion containing the 592 bp TNFRSF1B cDNA fragment from human monocytes, including three of the four optimized siRNA target sequences comprising the ON-TARGET plus SMART pool against TNFRSF1B. (B) The 592 bp TNFRSF1B cDNA fragment was cloned within the NheI and SalI restriction sites (highlighted in yellow boxes) within the pmirGLO vector. The primers used for the amplification are highlighted in red, and the three siRNA target sequences are highlighted in blue, orange, and magenta. (C) Luminescence FC values of 6CFSMEo- cells co-transfected with the ON-TARGET plus SMART siRNA pool against TNFRSF1B and with the pmirGLO-TNFRSF1B, compared to 6CFSMEo- cells co-transfected with the control siRNA(C-) and pmirGLO-TNFSRF1B. Data are expressed as means ± SD. n = 2, with three technical replicates/each. **p* <0.05 compared to the control; Figure S2. TNF signaling pathway induction in airway epithelial cells under oxidative stress conditions. A schematic representation of the KEGG TNF pathway (hsa04668). Circled in red are the TNF pathway transcripts that have been found to be involved in oxidative stress in airway epithelial cells through RNAi screening (Table 2). Highlighted in blue is the TNFR2 upstream interaction with one principal ligand: the soluble LT-α homotrimer, upregulated in airway epithelial cells under oxidative stress. Framed in magenta are the main cell death outcomes (apoptosis and necroptosis) resulting from the activation of the TNF pathway in airway epithelial cells under oxidative stress. TNF receptor crosstalk involves their association with common adaptor proteins such as TRAF2, initiating the recruitment of other signal transducers; Table S1: Putative transcript targets involved in oxidative stress after BLAST analysis considering the 167 siRNAs set derived from genome-wide RNAi screening in cystic fibrosis airway epithelial cells.
