# Supplementary material for: TNFRSF1B Signaling Blockade Protects Airway Epithelial Cells from Oxidative Stress
Source: Antioxidants (Basel). 2024 Mar 18;13(3):368. doi: 10.3390/antiox13030368 (PMC10968085; doi:10.3390/antiox13030368)
Supplement: Supplementary file 1 [file antioxidants-13-00368-s001.zip › antioxidants-2834720-supplementary/Figure S1 and S2.pdf]

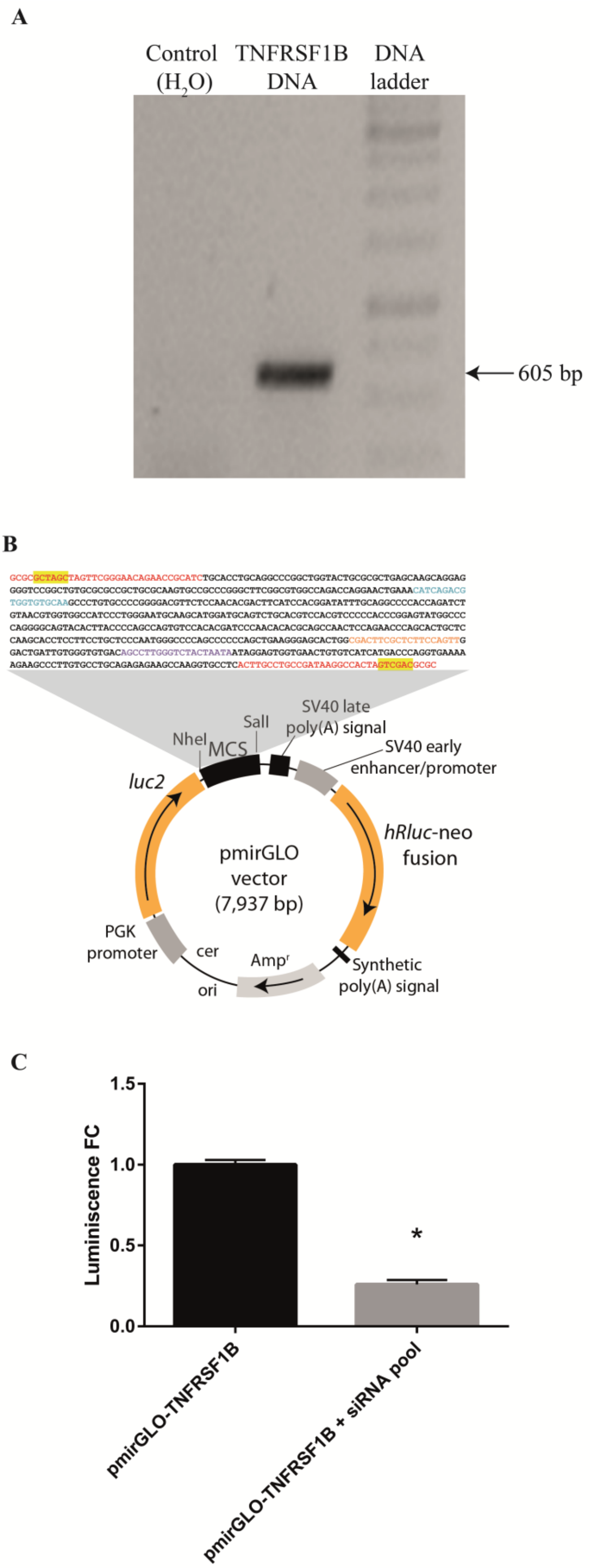


**Figure S1.** Optimized siRNAs induce efficient RNAi-mediated knock-down of TNFRSF1B. (A) PCR-mediated amplification of a 605 bp DNA portion

containing the 592 bp TNFRSF1B cDNA fragment from human monocytes, including 3 of the 4 optimized siRNA target sequences comprising the ON-TARGET plus SMART pool against TNFRSF1B. (B) The 592 bp TNFRSF1B cDNA fragment was cloned within the NheI and SalI restriction sites (highlighted in yellow boxes) within pmirGLO vector. The primers used for the amplification are highlighted in red, and the 3 siRNA target sequences are highlighted in blue, orange, and magenta. (C) Luminescence FC values of 6CFSMEo- cells co-transfected with the ON-TARGET plus SMART siRNA pool against TNFRSF1B and with the pmirGLO-TNFRSF1B, compared to 6CFSMEo- cells co-transfected with the control siRNA(C-) and pmirGLO-TNFSRF1B. Data are expressed as means ± SD. n=2, with 3 technical replicates/each. *p <0.05 compared to the control.

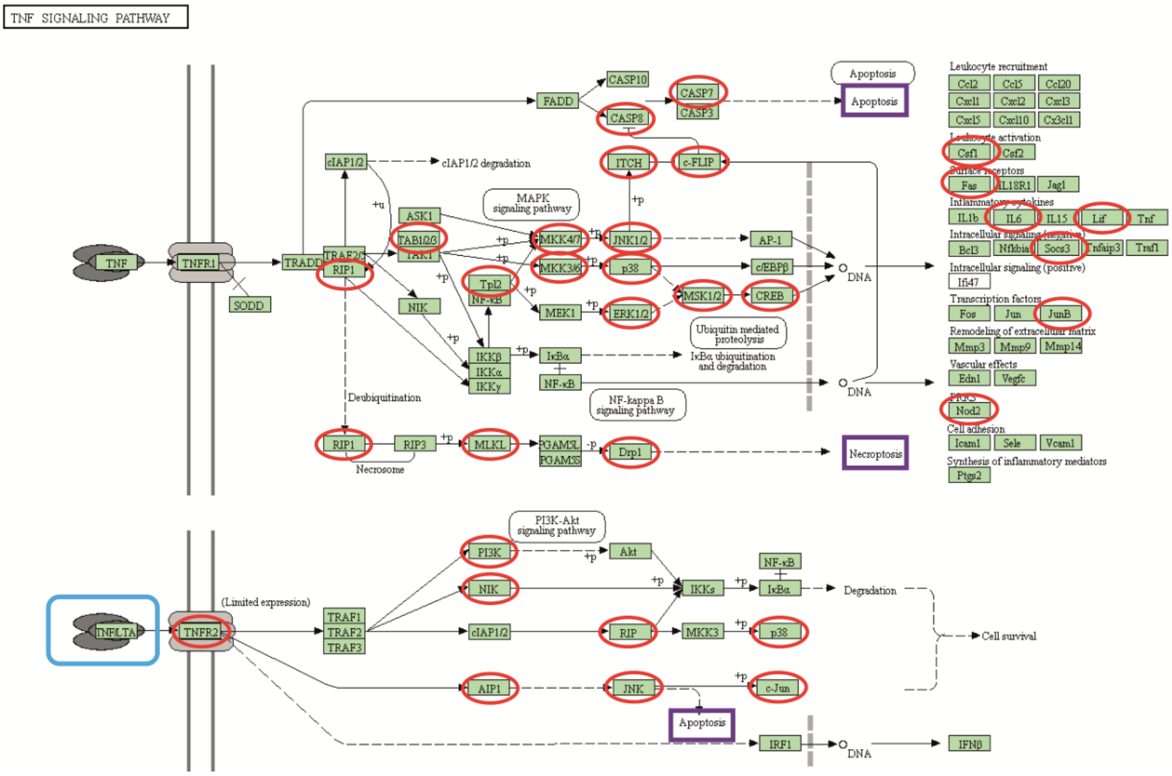


Figure S2. TNF signaling pathway induction in airway epithelial cells under oxidative stress conditions. Schematic representation of the KEGG TNF pathway (hsa04668). Circled in red are the TNF pathway transcripts that have been found involved in oxidative stress in airway epithelial cells through RNAi screening (Table 2). Highlighted in blue is the TNFR2 upstream interaction with one principal ligand: the soluble LT-α homotrimer, upregulated in airway epithelial cells under oxidative stress. Framed in magenta are the main cell death outcomes (apoptosis and necroptosis) resulting from the activation of the TNF pathway in airway epithelial cells under oxidative stress. TNF receptor cross talk involves their association with common adaptor proteins such as TRAF2, initiating the recruitment of other signal transducers
